# A definition of global oral health: An expert consensus approach by the Consortium of Universities for Global Health’s Global Oral Health Interest Group

**DOI:** 10.1080/16549716.2020.1814001

**Published:** 2020-09-03

**Authors:** Brittany Seymour, Zak James, Deepti Shroff Karhade, Jane Barrow, Alessio Pruneddu, Nina Kay Anderson, Peter Mossey, Task Force for the Definition of Global Health

**Affiliations:** a Global Health Discipline Director, Harvard School of Dental Medicine, Boston, MA, USA; b Kintegra Health, Charlotte, NC, USA; c Chapel Hill Adams School of Dentistry, University of North Carolina, Chapel Hill, NC, USA; d Associate Dean, Global and Community Health, Harvard School of Dental Medicine, Boston, MA, USA; e Department of Anthropology, University College London, Manchester, UK; f Department of Orthodontics and Pediatric Dentistry, Stony Brook School of Dental Medicine, Stony Brook, NY, USA; g Craniofacial Development, Associate Dean for Internationalisation, School of Dentistry, University of Dundee, Nethergate, Dundee, Scotland, UK; h Global Oral Health Interest Group, Consortium of Universities for Global Health, Boston, MA, USA

**Keywords:** Dr. Raman Preet, Umeå University, Global oral health, oral health, definitions

## Abstract

Definitions can generate actionable consensus for a given subject matter by resolving important differences in philosophy and best practices and by streamlining activities for a stronger strategic direction. Interest in the global dimensions of oral health, a generally neglected area of global health, is growing; yet, no previously published research has defined the term ‘global oral health.’ As such, the Global Oral Health Interest Group of the Consortium of Universities for Global Health determined a need for an introductory definition of ‘global oral health’ to guide program planning, implementation, and evaluation. With the oversight of an expert senior Task Force for the Definition of Global Oral Health, we employed a mixed-methods approach using the more common expert consensus-building Delphi technique combined with the lesser utilized Q methodology. This approach allowed us to identify the interconnectedness of global oral health themes and integrate multiple, seemingly disparate, topics into a single streamlined concept. Our resulting definition is as follows: *Global Oral Health aims for optimal oral health for all people and elimination of global health inequities through health promotion, disease prevention, and appropriate oral care approaches that consider common determinants and solutions and acknowledge oral health as part of overall health*. The purpose of this short communication is to generate a narrative around our proposed definition of global oral health to support establishing guidelines and developing best practices for academic global oral health programs, policies, and practices that aim to achieve a goal of oral health globally.

## Background

Definitions are common in order to generate actionable consensus for a given subject matter. They identify and resolve important differences in philosophy, best practices, and action in order to streamline efforts and determine strategic direction [1–3]. For example, an expert committee in Canada convened to develop a definition of ‘global health’ to support a strategic plan for Canada’s role in global health and development; the expert panel agreed that a formal definition was essential in laying the foundation for a cohesive and clear strategy moving forward [1]. FDI World Dental Federation published a new definition of ‘oral health’ in 2016, and different than the World Health Organization definition [4]. FDI determined that in order to *measure* oral health, a common definition must first be established to bring stakeholders together, define best practices, and determine priority-setting strategies for achieving it [2].

Oral health is a generally neglected area of health, both on national and global levels, and interest in the global dimensions of oral health has grown [5,6,7,8,9]. No previously published research has defined global oral health; thus, the term ‘global oral health’ is used differently by different professional communities. Global health and international health policy stakeholders tend to relate the term to the global burden of oral diseases, the existing inequalities, and possible policy solutions. The dental education community often associates the term with aspects of teaching, service learning, and outreach in the broadest sense. As such, the Global Oral Health Interest Group (GOHIG) [10] of the Consortium of Universities for Global Health (CUGH) [11] determined a need, as a complement to its Global Oral Health Competency Matrix [12], for a suggested definition of ‘global oral health’ to guide program planning, implementation, and evaluation. The definitions of ‘global health’ and ‘health’ share commonalities and distinctions that allow them to function together toward a goal of health globally. Similarly, the purpose of this communication is to generate a narrative around a shared definition of global oral health to support establishing guidelines and developing standards for academic global oral health programs and practices that aim to achieve a goal of oral health globally. The Harvard Faculty of Medicine IRB Office of Human Research Administration determined this study protocol IRB17-0992 as exempt.

## Methods

In attempting to ascertain the broad, qualitative nature of disparate perspectives concerning a definition of global oral health, we employed a mixed-methods approach using the more common expert consensus-building Delphi technique [13], combined with the lesser utilized Q methodology. A strength of the Q method is its ability to identify relationships and interconnectedness of a given topic’s themes; this makes it particularly suitable for developing a definition, which requires integrating multiple, sometimes disparate, topics into a single streamlined concept [14]. The consensus approach took place in three phases and was overseen by a senior expert Task Force for the Definition of Global Oral Health, selected by the GOHIG membership. Dispersed globally, the Task Force consisted of 10 expert professionals in global health, oral health, biomedical sciences, and other related disciplines with a stake in the development of global oral health terminology.

Beginning with the Task Force members, and using a snowballing technique for broader representation of expert opinion and philosophy, we identified an additional 12 professionals from other interested organizations beyond CUGH (totaling 22 expert respondents) to participate in the surveys (Table 1).

**Table 1.** Demographics of our 22 consensus experts for defining Global Oral Health.

WHO Region	Affiliation	Dental/Nondental
African (Region 1): 1 Americas (Region 2): 18 European (Region 4): 1 SE Asia (Region 3): 2	Academic Institution: 13 American Association of Public Health Dentistry: 5 American Dental Association: 7 American Dental Education Association: 7 Consortium of Universities for Global Health: 9 FDI World Dental Federation: 1 International Association for Dental Research: 7 World Health Organization: 1	14 dentists 8 nondentists

The Phase I survey was designed to be intentionally broad, allowing the expert participants liberty for creative and unrestricted responses to generate sufficient data for later, more structured survey development designed to yield a convergence of opinion. This phase used a semi-structured brief Qualtrics survey instrument in which respondents were prompted to answer a series of five open-ended questions by providing statements about global oral health. The questions were developed de novo and were designed to both capture a broad array of expert opinion statements related to ‘global oral health,’ but also to remain concise enough to mitigate excessive and cumbersome data collection.

**Figure 1.**
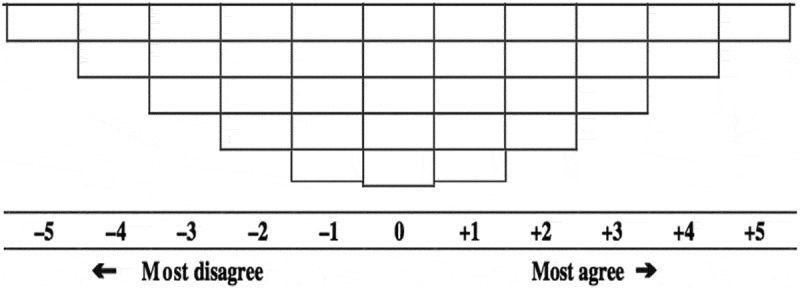
Q Sort boxes totaling 35, one for each statement presented in the Phase II survey. Respondents were asked to drag and drop each statement into a box that corresponded with their level of agreement with that statement, ranging from ‘most disagree to most agree.’ Only one statement per box was permitted and all statements had to be sorted into a box.

Phase II utilized the Q-methodology sorting approach to quantify participants’ level of agreement with the results from Phase I. A Q-set of statements was generated from Phase I, and participants were asked to sort them according to their level of agreement with each one, ranging from ‘Most Agree’ to ‘Least Agree.’ The Q-sort followed a quasi-normal-forced choice distribution, in which the boxes at the tail of the distribution accepted the fewest statements, while the middle group (Undecided) permitted the greatest number of sorted responses. Each box was scored, ranging from −5 (Least Agree), to +5 (Most Agree), see Figure 1. Data were collected using the QSortouch [15], an electronic software tool specifically designed to work with Q-methodology and Q-sort techniques. Significant statements were accordingly clustered into three consensus narratives, defined as ‘factors,’ which served as precursors to a possible definition of ‘global oral health.’

In Phase III, respondents were asked to rank the three factors in order of preference. A smaller set of individually coded statements that were statistically determined to generate adequate consensus from the *Q* methodology round were isolated; the final survey also asked respondents to rank these statements in order of preference. Factor arrays were performed to validate which statements were in consensus with which factors. For each factor, we recorded the number of participants who loaded onto (aligned with) it based on their ranking results, the Eigenvalues (significance of the factor based on respondent ranking), variance explained (number of Q sorts from Phase II that are closely aligned with the Factor), and reliability (related to Phase III statement ranking). Grounded in the data, and based on statistical consensus generated from the factor and statement rankings, the research team drafted a definition of global oral health.

## Results

The Phase I round of surveys yielded 35 statements (Table 2), a standard number for an effective Q method approach. Factor array results are located in Table 3; these list the number of participants who loaded onto each factor, the significance of the factor based on Phase III ranking, and variance explained. Combined analysis of Phase III statement and factor rankings showed greatest preference for factor 1, with some elements of factor 2. Taken together, with respondent validation through open-ended comments from the surveys and final discussion with the Task Force, the recommended definition of ‘global oral health’ from this expert consensus approach is as follows:

Global Oral Health aims for optimal oral health for all people and elimination of global health inequities through health promotion, disease prevention, and appropriate oral care approaches that consider common determinants and solutions and acknowledge oral health as part of overall health.

**Table 2.** Final *Q* set of 35 expert statements generated from the Phase I Delphi survey related to the overarching question, ‘What is global oral health?’.

1	An act with regard to oral health that can be applied globally
2	Encompasses oro-facial pathologies throughout the world
3	Indicator for oral health that is applicable for all nations
4	A set of activities designed to improve the oral health of communities around the world
5	Compares disparities and oral health needs between different countries and regions
6	The management of oral diseases globally
7	Diffusion of knowledge pertaining the oral cavity and its role in nutrition and hydration
8	A set of activities designed to educate the workforce to provide preventive and therapeutic care for the oral health of communities around the world
9	Explores the concept of health through the examination of disease
10	Think globally, act locally
11	Is a state of physical, mental, and social wellbeing
12	Refers to the prevailing oral disease regional distribution on the global scale
13	Implies worldwide coverage of the human population
14	Is a perspective on disease and wellness related to oro-dental conditions
15	Is oral health status all over the world which is measured using calibrated oral health indicators
16	Has changed over time and continues to evolve, taking on different meaning under different lenses and contexts
17	Oral health equally for all humans
18	Is based on community leadership and partnership
19	Is grounded in principles of ethics and sustainability
20	Acknowledges that health challenges and solutions transcend borders
21	Is essentially related to systemic health
22	Is an area of healthcare that includes direct patient care, epidemiology, research, and dental workforce training to evaluate and improve oral health in populations globally
23	Involves the policies in oral health promotion and disease prevention
24	Must imply physiological and psychological concepts in addition to the disease state of the oral cavity
25	Strives to connect the dental professional into a larger global health movement
26	Requires knowledge of global trends, the development agenda, and how they apply to local context
27	Is full understanding of the global burden of oral and craniofacial diseases and conditions
28	Is working to promote optimal oral health for all people
29	Facilitation of access to preventive and restorative care that seeks to promote oral and systemic function and quality of life
30	Refers to the global picture of oral diseases, the oral disease burden on populations, preventive and promotive methods that have success in world populations
31	Is a mass movement around the globe towards one vision, one message on oral health
32	Is elimination of global oral health inequities
33	Focuses on health promotion, prevention, and integration into existing programs
34	Is a multidisciplinary domain including dentistry, medicine, nutrition, epidemiology, and other specialties
35	Recognizes that the ramifications and the causes of oral diseases extend beyond the oral cavity

**Table 3.** Factor analysis yielding general factor characteristics.

General factor characteristics				
*F*	No. of loading	Eigenvalues	Var. explained	Reliability
Factor 1	8	4	18	0.97
Factor 2	8	4	18	0.97
Factor 3	4	2.4	11	0.94

## Discussion

The GOHIG definition for global oral health shares several common themes with literature reports on oral health in a global context, including integrating oral health and primary care and streamlining efforts for preventing and controlling oral diseases and other NCDs [16,17]. The 2019 Lancet Series on Oral Health has a strong focus on equity, and key messages from this series are represented in our proposed definition, including moving from treatment-dominated approaches to prevention and promotion, integrating oral health into health care and overall health and well-being, addressing common risk factors for NCDs and social determinants of health, and identifying appropriate treatment within the frameworks of universal health coverage and the Sustainable Development Goals [5,6]. Similar themes are reflected in the key priorities of the Lancet Commission on Oral Health, formed in early 2020 [18].

Related definitions reinforce but also differentiate themselves from our recommended definition of global oral health. Glick et al., in defining oral health, emphasize its relationship to overall health as well. Review of their approach illustrates that ‘oral health’ can be viewed as a state of being [2], versus ‘global oral health’ as an actionable approach to achieving oral health at the global level; the two are inextricably related in that the latter aims to realize the former through action. Reports on defining ‘global health’ recognize impacts of globalization on population health, including health risks and determinants, and surpassing international or geographic designations to assume a global conceptualization [1,19]. Koplan et al.’s definition of global health focuses on broad determinants that directly or indirectly impact health and transcend boundaries. Their definition also demands interdisciplinary cooperation at the global level, embraces both clinical and preventive measures with emphasis on the latter, and shares a strong objective of equity [3]. A definition of ‘dental public health’ places emphasis not on clinical intervention but rather focuses on population-level approaches as a specialty of dentistry, while also recognizing oral health as an essential component of general health and quality of life. A difference between dental public health and global oral health is that the former tends to have a predominant focus on a single society/nation rather than a global application, while the latter builds connectivity and recognizes commonality across borders [20]. Another report on defining global health reiterates the move from nationally based efforts in traditional public health paradigms toward collaborative transnational efforts, which include two or more countries [21]. Our suggested definition is the result of a small-scale expert consultation within a specific working group (GOHIG CUGH), and thus did not aim for representativeness. Our chosen mixed methodology assisted in supporting the development of a reliable definition that can serve as a starting point for global discussion and lead to the next steps for further work and study. We recognize the necessity to continue toward a truly global and inclusive approach to all efforts underway to improve health globally.

## Conclusion

This study presents the first-ever definition for ‘global oral health.’ Definitions are important vehicles for resolving differences in philosophy, strategy, and action and for streamlining priority-setting and determining best practices. The proposed definition is well aligned with important public health and contemporary global health frameworks and complements the GOHIG Global Oral Health Competency Matrix. We hope this proposed definition stimulates international discussion among stakeholders, which in itself is a key aspect of the process towards alignment and consensus building for academic global oral health programs, policies, and practices.

## Paper context

Definitions, such as for ‘global health’ and ‘oral health,’ can generate actionable consensus and resolve philosophical differences. Interest in the global dimensions of oral health is rising; yet, no previously published research has defined ‘global oral health.’ We identified the interconnectedness of global and oral health themes and integrated topics into a streamlined complementary definition for ‘global oral health.’ This definition can stimulate consensus building for academic global oral health programs, policies, and practices.
